# Computed tomography texture analysis to facilitate therapeutic decision making in hepatocellular carcinoma

**DOI:** 10.18632/oncotarget.7467

**Published:** 2016-02-17

**Authors:** Meng Li, Sirui Fu, Yanjie Zhu, Zaiyi Liu, Shuting Chen, Ligong Lu, Changhong Liang

**Affiliations:** ^1^ Southern Medical University, Guangzhou, China; ^2^ Department of Radiology, Guangdong General Hospital, Guangdong Academy of Medical Sciences, Guangzhou, China; ^3^ Department of Interventional Oncology, Guangdong Provincial Cardiovascular Institute, Guangdong General Hospital, Guangdong Academy of Medical Sciences, Guangzhou, China; ^4^ Shenzhen Institutes of Advanced Technology, Shenzhen, China

**Keywords:** hepatocellular carcinoma, texture analysis, computed tomography, liver resection, transcatheter arterial chemoembolization

## Abstract

This study explored the potential of computed tomography (CT) textural feature analysis for the stratification of single large hepatocellular carcinomas (HCCs) > 5 cm, and the subsequent determination of patient suitability for liver resection (LR) or transcatheter arterial chemoembolization (TACE). Wavelet decomposition was performed on portal-phase CT images with three bandwidth responses (filter 0, 1.0, and 1.5). Nine textural features of each filter were extracted from regions of interest. Wavelet-2-H (filter 1.0) in LR and wavelet-2-V (filter 0 and 1.0) in TACE were related to survival. Subsequently, LR and TACE patients were divided based on the wavelet-2-H and wavelet-2-V median at filter 1.0 into two subgroups (+ or −). LR+ patients showed the best survival, followed by LR-, TACE+, and TACE-. We estimated that LR+ patients treated using TACE would exhibit a survival similar to TACE- patients and worse than TACE+ patients, with a severe compromise in overall survival. LR was recommended for TACE- patients, whereas TACE was preferred for LR- and TACE+ patients. Independent of tumor size, CT textural features showed positive and negative correlations with survival after LR and TACE, respectively. Although further validation is needed, texture analysis demonstrated the feasibility of using HCC patient stratification for determining the suitability of LR *vs*. TACE.

## INTRODUCTION

Identification and quantification of tumor heterogeneity by computed tomography (CT) textural analysis shows promise in enhancing prognostic accuracy and facilitating therapeutic decision making. Such advances are particularly important for diseases such as liver cancer, which is the second and sixth most frequent cause of cancer related-death in men and women, respectively, with hepatocellular carcinoma (HCC) most common [1]. According to the Barcelona Clinic Liver Cancer (BCLC) staging system, the diameter of a single HCC may not be a contraindication for liver resection (LR) [2-5]. However, most Asian patients with HCC have diseased liver parenchyma, such as hepatitis B virus infection and/or hepatitis B virus-related cirrhosis, and LR in this population is therefore associated with a high risk of complications [6]. This consideration may alter therapy-based decision-making in cases of single HCCs > 5 cm, particularly for potential LR candidates [7, 8]. Asymptomatic patients with a solitary HCC without vascular invasion or extrahepatic spread and with well-preserved liver function could also be considered for transcatheter arterial chemoembolization (TACE) [3, 5, 9]. Thus, to assess whether a patient scheduled to undergo LR would be better suited for TACE and vice versa, reliable prognostic markers for patient stratification are needed.

Proposals for a subclassification system for BCLC stage B tumors have emerged in recent years. One study proposed a stratification system aimed toward tailoring therapeutic interventions based on both the evidence available to date and expert opinions [10]. Another study suggested taking the Child-Pugh score and liver transplantation status into account [11]. In clinical practice, the decision to treat with LR or TACE is made using a combination of clinical symptoms, laboratory test results, and pathological biomarkers, whereas the CT images routinely acquired during treatment and follow-up are largely overlooked. Conventional assessment of tumor size and enhancement of cross-sectional images are far from satisfactory in the determination of an appropriate therapeutic strategy, due to insufficient imaging of the inherent properties of the tumor and interobserver variability in image interpretation.

Radiomics is an emerging research field that aims to utilize the full potential of medical imaging [12]. This includes texture analysis, which is assumed to reflect tissue heterogeneity [13-17]. Heterogeneity is a well-recognized feature reflecting alterations in tissue patterns, likely occurring due to cell infiltration, abnormal angiogenesis, microvasculature and necrosis [18-20]. One study suggested an association between image traits, including textural features, and underlying gene expressions in HCC [21]. Another stated that the radiomic signature could be transferred from lung to head-and-neck cancer, suggesting that this signature identifies a general prognostic tumor phenotype [12]. In fact, texture analysis has shown feasibility in the differential diagnosis of liver cancers [22], hepatic fibrosis detection/staging [23, 24], and prediction of postoperative hepatic insufficiency [25].

In this study, we explored texture analysis as a prognostic and patient stratification approach in the determination of the appropriate therapeutic option, LR or TACE, for patients with single large HCCs. Herein, two questions were raised: (1) are the textural parameters of the primary tumor, calculated from baseline CT, related to prognosis? (2) Does texture analysis have the potential to provide an additional view for treatment modification between LR and TACE?

## RESULTS

### Patients

A total of 130 patients (86 and 44 treated by LR and TACE, respectively) were retrospectively included for texture analysis. Of these, 106 (81.5%) patients had disease progression, and 96 (73.8%) patients died by the study end date. There were no significant differences in the patient baseline demographics and characteristics (Table 1). All texture features, calculated from two sets of regions of interest (ROIs), showed excellent agreement (ICC value, 0.799-0.999).

**Table 1:** Patient baseline demographics and characteristics

	All (*n*=130)	LR (*n* =86)	TACE (*n* =44)	*P*
**Age (years)**	57 (20–84)^*^	56(30–84) ^*^	59 (20–81) ^*^	0.125
**Sex (n)**				0.425
**Male**	114	74	40	
**Female**	16	12	4	
**Body mass index (kg/m**^2^**)**	23(15–32)^*^	23 (17–31) ^*^	24 (15–32) ^*^	0.304
**Hepatitis infection (n)**				0.203
**HBV**	88	55	33	
**Negative**	42	31	11	
**Child–Pugh class (n)**				0.209
**A**	125	84	41	
**B**	5	2	3	
**Performance status**				
**0**	118	81	37	0.105
**1**	12	5	7	
**BCLC stage**				0.742
**AB (without VI)**	114	76	38	
**C (with VI)**	16	10	6	
**Cirrhosis**				0.244
**Yes**	92	58	34	
**No**	38	28	10	
**Maximum diameter (mm)**	80 (51–187) ^*^	85(51–150) ^*^	76(52–187) ^*^	0.821
**Albumin (g/L)**	36 (23–48)^*^	36 (27–48)^*^	36 (23–43)^*^	0.273
**Total bilirubin (μmol/L)**	18 (6–47)^*^	17 (6–32)^*^	19 (10–47)^*^	0.454
**Prothrombin time (sec)**	14 (12–16)^*^	14 (12–15)^*^	14 (12–16)^*^	0.418
**ALT (U/L, n)**	38(10–566)^*^	38(10–236)^*^	38(17–566)^*^	0.509
**AFP level (ng/ml)**				0.369
**<25**	47	32	15	
**25–400**	45	32	13	
**>400**	38	22	16	
**Differentiation(n)**				
**Unknown**	44	0	44	
**Moderate**	32	32	-	
**Moderate-poor**	11	11	-	
**Poor**	43	43	-	
**Microvascular invasion(n)**				
**Unknown**	44	0	44	
**Negative**	40	40	-	
**Positive**	46	46	-	

* Median (range) for data without normal distribution.

LR: liver resection; TACE: transcatheter arterial chemoembolization; BCLC: Barcelona Clinic Liver Cancer; VI: vascular invasion; ALT: alanine aminotransferase; AFP: Alpha fetoprotein;

### Cox regression and Kaplan-Meier analysis for LR and TACE

For candidate clinical and imaging variables, univariate analysis showed that corona (*P* = 0.057) was the only variable with a *P* value < 0.10 in LR, whereas in TACE, none of the variables showed significant differences (Table 2). For textural features, nine and 21 features in the LR and TACE groups, respectively, were identified as statistically significant (Table S1).

**Table 2:** Univariate Cox regression of clinical variables and radiological features for overall survival in LR and TACE group

	LR			TACE		
Factors	*n*	HR (95% CI)	*P*	*n*	HR (95% CI)	*P*
**Sex** female	12	reference	0.843	4	reference	0.269
**male**	74	1.103 (0.419–2.904)		40	30.802 (0.071–13345.010)	
**Age**	86	0.981 (0.952–1.012)	0.225	44	1.016 (0.987–1.045)	0.281
BCLC stage* C	10	Reference	0.117	6	Reference	0.163
AB	76	0.427 (0.147–1.237)		38	0.380 (0.097–1.478)	
**Maximum diameter**	86	1.005 (0.994–1.016)	0.337	44	0.998 (0.987–1.008)	0.660
**Cirrhosis** positive	28	Reference	0.388	10	Reference	0.425
**negative**	58	0.704 (0.318–1.561)		34	0.753 (0.328–1.624)	
**Child-Pugh Class** B	2	Reference	0.940	3	Reference	0.590
A	84	1.080 (0.146–7.996)		41	1.518 (0.333–6.921)	
**Hepatitis** HBV	31	Reference	0.557	11	Reference	0.321
**negative**	55	1.256 (0.587–2.689)		33	0.570 (0.188–1.729)	
**AFP** (ng/ml) >400	22	Reference	0.522	16	Reference	0.802
<25	32	1.018 (0.394–2.633)		15	0.906 (0.313–2.624)	
25∼400	32	1.593 (0.603–4.209)		13	1.349 (0.412–4.420)	
**Post-TACE** yes	44	Reference	0.791	-	All received post-TACE	-
**no**	42	0.904 (0.429–1.906)				
**Post-ablation** yes	8	Reference	0.164	16	Reference	0.709
**no**	78	4.139 (0.560–30.580)		28	1.187 (0.482–2.924)	
**Capsule** integral	14	Reference	0.396	14	Reference	0.125
**absence**	9	1.129 (0.206–6.181)		14	3.475 (1.049–11.510)	
**not integral**	63	1.955 (0.667–5.727)		16	1.917 (0.619–5.933)	
**Shape** invasive	29	Reference	0.814	23	Reference	0.108
**non-invasive**	57	0.911 (0.419–1.982)		21	0.469 (0.186–1.182)	
**Corona†** positive	40	Reference	0.057	15	Reference	0.367
**negative**	46	0.478 (0.224–1.021)		29	0.650 (0.255–1.657)	
**Mosaic** positive	67	Reference	0.144	31	Reference	0.150
**negative**	19	0.485 (0.184–1.280)		13	2.032 (0.773–5.336)	
**Node in Node**		All negative			All negative	
**Enhance region** >75%	67	Reference	0.894	38	Reference	0.223
0%-25%	-	-		-	-	
25%–50%	5	0.704 (0.095–5.235)		2	2.478 (0.300–20.433)	
50%–75%	14	1.158 (0.438–3.065)		4	3.720 (0.768–18.020)	
**Differentiation** poor	43	Reference	0.713		Not available in TACE group	-
moderate	32	1.028 (0.541–1.863)				
moderate-poor	11	0.725 (0.355–1.748)				
**MI** positive	46	Reference	0.452		Not available in TACE group	-
negative	40	0.703 (0.297–1.742)				

Dash indicated no data identified; *in this study, presence of vascular invasion was represented by BCLC stage (B: absence; C, presence); †factors in the univariate analyses with a *P* <0.10 entered multivariate analyses; # All LR patients had assessments of microvascular invasion, whereas it was not available in TACE patients.

LR: liver resection; TACE: transcatheter arterial chemoembolization; HR: hazard ratio; BCLC: Barcelona Clinic Liver Cancer; AFP: alpha fetoprotein; MI: microvascular invasion;

Multivariate Cox models showed that only wavelet-2-H (filter 1.0) in LR and wavelet-2-V (filter 0 and 1.0) and wavelet-3-D (filter 1.5) in TACE were significantly correlated with overall survival (OS) (Table 3).

**Table 3:** Multivariate Cox regression for overall survival^^^

Group	Factors	HR (95% CI)	*P*
**LR**		**Filter=0**	
		**-**	
		**Filter=1.0**	
	Wavelet-2-H	0.836 (0.700–0.998)	0.047
		**Filter=1.5**	
		**-**	
**TACE**		**Filter=0**	
	Wavelet-2-V	1.120 (1.012–1.239)	0.029
		**Filter=1.0**	
	Wavelet-2-V	1.209 (1.025–1.426)	0.024
		**Filter=1.5**	
	Wavelet-3-D	3.146 (1.102–8.979)	0.032

**Figure 1:**
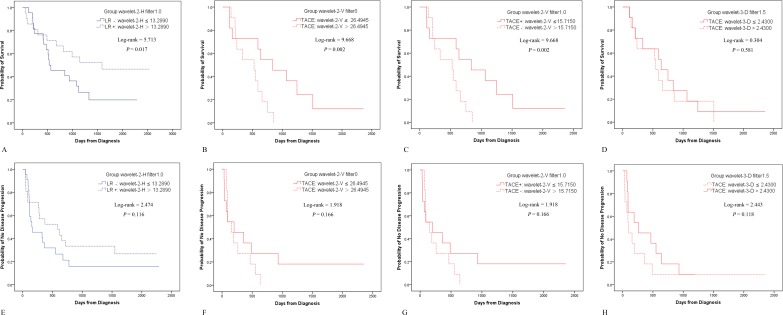
Kaplan-Meier analyses for LR and TACE When separated by wavelet-2-H at filter 1.0 and wavelet-2-V at filter 0 and 1.0, respectively, there were significant differences in OS **A.**, **B.** & **C.** but not in TTP **E.**, **F.** & **G.** If separated by wavelet-3-D (filter 1.5), neither OS nor TTP showed a significant difference **D.** & **H.**

Dash indicated no data identified; ^^^6 separate multivariate cox regression were performed, only variables with a statistical significance were listed (no clinical variables or radiologic features were identified after multivariate Cox regression);

LR: liver resection; TACE: transcatheter arterial chemoembolization; HR: hazard ratio;

Separated by the above-identified four textural parameters in LR and TACE, OS differed significantly for each feature whereas time to progression (TTP) did not (Figure 1 & Table 4). There were no significant differences in patient demographics and characteristics.

**Table 4:** Kaplan-Meier method and Log-rank tests

Treatment	Group	OS		TTP	
		mOS # (95%CI)	*P*	mTTP# (95%CI)	*P*
**LR**	**Wavelet-2-H (filter 1.0)**				
	LR-	533 (262–804)	0.017*	153 (0–323)	0.116
	LR+	Less than half died		589 (228–950)	
**TACE**	**Wavelet-2-V (filter 0)**				
	< 26.4945	837 (475–1199)	0.002 *	199 (0–420)	0.166
	> 26.4945	525 (287–763)		154 (78–230)	
	**Wavelet-2-V (filter 1.0)**				
	TACE+	837 (475–1199)	0.002 *	199 (0–420)	0.166
	TACE-	525 (287–763)		154 (78–230)	
	**Wavelet-3-D (filter 1.5)**				
	≤ 2.4300	637 (318–956)	0.581	94 (30–158)	0.118
	> 2.4300	548 (278–818)		260 (0–547)	

# Unit: days

* with a statistical difference

LR-: LR patients with wavelet-2-H (filter 1.0) <13.2890; LR+: LR patients with wavelet-2-H (filter 1.0) >13.2890; TACE-: TACE patients with wavelet-2-V (filter 1.0) < 15.7150; TACE+: TACE patients with wavelet-2-V (filter 1.0) >15.7150; OS: overall survival; TTP: time to progression; LR: liver resection; TACE: transcatheter arterial chemoembolization.

### Kaplan-Meier analyses and cox regression for subgroups

**Figure 2:**
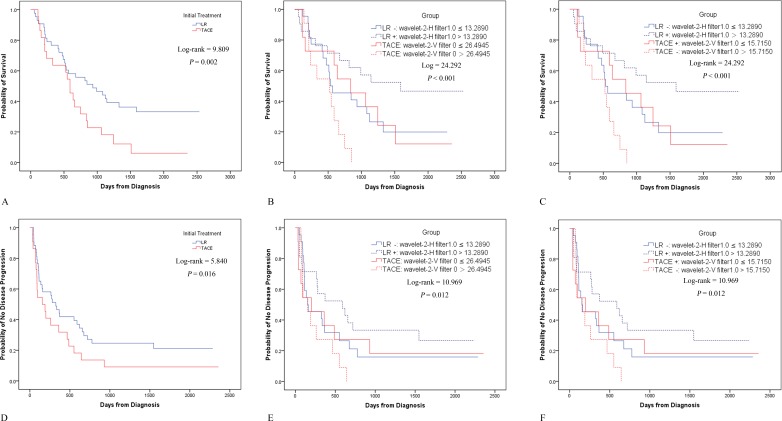
Kaplan-Meier analyses for various subgroups Before subgrouping, OS **A.** and TTP D. showed significant differences between LR and TACE. Upon subgrouping by wavelet-2-H (filter 1.0) in LR and by wavelet-2-V (filter 0) in TACE, LR+ was associated with the best OS, followed by LR- and TACE+; TACE- had the poorest OS **B.** TTP also showed significant differences, with the TTP of LR+ and LR- being equal to that of TACE+, but better than that of TACE- E. Similar results for OS **C.** and TTP F. were noted if LR was separated by wavelet-2-H (filter 1.0) and TACE was separated by wavelet-2-V (filter 1.0).

Without detailed subgrouping, patients in the LR group showed better OS (χ^2^ = 9.809, *P* = 0.002) and TTP (χ^2^ = 5.840, *P* = 0.016) than those in the TACE group (Figure 2A & 2D). Subsequently, patients were divided into four subgroups: LR-, LR+, TACE+, and TACE-. Since the LR group had longer TTP (some patients were without recurrence), this group received fewer sessions of subsequent TACE and ablation than patients in the TACE group. As a result, 44 of 86 (51.2%) LR patients (median number of sessions, 1; range, 0-9) and 44 of 44 (100%) TACE patients (median number of sessions, 3; range 1-15) received subsequent TACE, while 9 of 86 (10.5%) LR patients (median number of sessions, 0; range, 0-4) and 16 of 44 (36.4%) TACE patients (median number of sessions, 0; range, 0-3) received subsequent ablation. There were no other differences in patient demographics and characteristics among the four subgroups.

OS showed a significant difference among the four subgroups (χ^2^ = 24.292, *P* < 0.001). Further pairwise comparisons showed that LR- *vs*. TACE- (χ^2^ = 8.229, *P* = 0.004), LR+ *vs*. TACE+ (χ^2^ = 4.425, *P* = 0.035) and LR+ *vs*. TACE- (χ^2^ = 21.880, *P* < 0.001) had significant differences in OS, whereas LR- *vs*. TACE+ (χ2 = 0.010, *P* = 0.920) did not (Figure 2C). Similar results were noted when LR was separated by wavelet-2-H (filter 1.0) and TACE was separated by wavelet-2-V (filter 0) (Figure 2B).

TTP also showed a significant difference between the four subgroups (χ2 = 10.969, *P* = 0.012). Further pairwise comparisons showed that LR - *vs*. TACE- (χ2 = 4.317, *P* = 0.038) and LR+ *vs*. TACE- (χ2 = 11.762, *P* = 0.001) had significant differences in the TTP, whereas LR- *vs*. TACE+ (χ2 = 0.034, *P* = 0.854) and LR+ *vs*. TACE+ (χ2 = 2.299, *P* = 0.129) did not (Figure 2F). Similar results were noted when LR was separated by wavelet-2-H (filter 0) and TACE was separated by wavelet-2-V (filter 0) (Figure 2E).

In all patients, for OS, univariate Cox regression showed that BCLC, corona, and subgrouping had *P*-values < 0.10, and the multivariate Cox regression models confirmed that subgrouping was the only factor that was significantly associated with OS (*P* = 0.012). For TTP, univariate Cox regression showed that the presence of a capsule, corona and subgrouping had *P*-values < 0.10, and the multivariate Cox regression models confirmed that the capsule was the only factor that was significantly associated with the TTP (*P* = 0.021).

These results indicate that LR+ was associated with the best survival, followed by LR- and TACE+ (*P* = 0.920 and 0.854 for OS and TTP, respectively, in LR- *vs*. TACE+), whereas TACE- was associated with the worst survival. Thus, the feasibility of texture features in patient stratification and determination of the most suitable therapy (LR or TACE) was partly confirmed; however, further validation was still considered necessary.

### Further validation

Since wavelet-2-V (filter 1.0) and wavelet-2-H (filter 1.0) were not normally distributed among the subgroups, Kruskal-Wallis *H* was used for further analysis.

**Figure 3:**
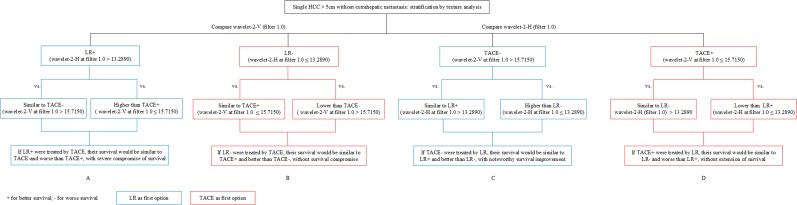
Schematic flow of further validation studies

First, wavelet-2-V (filter 1.0) was compared between LR+ and TACE+, as well as between LR+ and TACE-. The results showed that the value of LR+ was similar to that of TACE- (median, 17.7020 *vs*. 18.3490, *P* > 0.999), but higher than that of TACE+ (median, 17.7020 *vs*. 12.8860, *P* < 0.001). Therefore, if LR+ patients are treated by TACE, their survival would be similar to that of the TACE- group and worse than that of the TACE+ group, with severe compromise of OS (Figure 3A).

Second, wavelet-2-V (filter 1.0) was compared between LR- and TACE+, as well as between LR- and TACE-. The results showed that the value of LR- was similar to that of TACE+ (median, 13.1050 *vs*. 12.8860, *P* > 0.999), but lower than that of TACE- (median, 13.1050 *vs*. 18.3490, *P* < 0.001). Therefore, if LR- patients are treated by TACE, their survival would be similar to that of TACE+ patients and better than that of TACE- patients, without compromise of OS (Figure 3B).

Third, we compared wavelet-2-H (filter 1.0) between TACE- and LR-, as well as between TACE- and LR+. We observed that TACE- and LR- showed a significant difference (median: 15.3530 *vs*. 11.8430, *P* < 0.001), whereas TACE- and LR+ did not (median: 15.3530 *vs*. 15.7260, *P* > 0.999). Therefore, if TACE- patients are treated by LR, their survival would be similar to that of LR+ patients and better than that of LR- patients, and their OS would be considerably improved (Figure 3C).

Lastly, we compared wavelet-2-H (filter 1.0) between TACE+ and LR-, as well as between TACE+ and LR+. We observed that TACE+ and LR+ showed a significant difference (median: 11.5270 *vs*. 15.7260, *P* < 0.001), whereas TACE+ and LR- did not (median: 11.5270 *vs*. 11.8430, *P* > 0.999). Therefore, if TACE+ patients are treated by LR, their OS would be similar to that of LR- patients and worse than that of LR+ patients, with no extension of survival (Figure 3D).

Accordingly, when the TACE group was separated by another prognostic indicator, wavelet-2-V (filter 0), similar results were consistently noted (Figure 2).

## DISCUSSION

In this study, we took six typical HCC subject image features and textural features into account to determine whether or not they could be used to assist in therapeutic decision-making and optimization. Corona and 29 textural parameters (nine in LR and 20 in TACE) had *P*­-values < 0.10 in the univariate Cox regressions for OS. Sequentially, multivariate Cox regressions and Kaplan-Meier analyses identified four parameters (one in the LR group and three in the TACE group) related to OS.

Filter 1.0 was the best filter, as it showed significant results in both the LR and TACE groups, which was consistent with the findings of published studies [15, 16]. The reason for this result might be that textural features at filter 0 tend to reflect radiologists’ impressions of image quality, which could be influenced by image noise. By using filters at larger scales (filter 1.0, 5 pixels), subjective bias might be alleviated, and underlying biologic heterogeneity could be enhanced [14].

In the subgroup comparisons, we noted that TACE would have severely compromised OS in LR+ patients, while LR would have considerably improved OS in TACE- patients; thus, LR should be recommended for these patients (Figure 3, blue part). On the other hand, TACE+ patients treated by LR would have no extension of survival, while LR- patients treated by TACE would not have their survival compromised; in these cases, TACE is recommended (Figure 3, red part).

Further validation showed that lower wavelet-2-H (filter 1.0) was simultaneously with lower wavelet-2-V (filter 0 and 1.0) and vice versa, which was consistent with the definition of wavelet-2-H and 2-V, both features were extracted from reconstructed images originating from the same decomposing level (level 2). Thus, single large HCCs (well-preserved liver function, no extrahepatic metastasis) with higher wavelet-2-H (filter 1.0) and wavelet-2-V (filter 0 and 1.0) are considered suitable for LR, whereas those with lower values might be recommended for TACE (Figure 3). Additionally, similar conclusions could also be drawn if separated by the receiver operating characteristic curve threshold (Figure S1 & S2).

Although BCLC stage C used to be considered a contradiction for both TACE and LR in HCC, recent studies showed that both TACE and LR could provide survival benefit [26-28]. Nevertheless, patient selection was crucial before LR or TACE. So, we included HCCs in BCLC stage C in our study. Furthermore, in clinical observations, sorafenib alone seldom led to necrosis in intrahepatic/extrahepatic lesions or shrinkage in thrombosis. Given that no more effective treatments are recommended by the BCLC staging system, BCLC stage C HCC patients would have significantly shorter survival times than stage B patients. However, in this study, patients in BCLC C stage only had thrombosis at bifurcations. In this situation, the branches involved could be removed by resection or embolized by TACE. Therefore, intrahepatic lesions and branch vascular invasion could be treated simultaneously. This might be one explanation for why the BCLC did not have prognostic value in this study. Nevertheless, our results should be considered as preliminary, and further study is warranted.

In HCC prognosis, tumor stage and accurate evaluation of the liver-function reserve need to be incorporated [5]. Thus, the presence of cancer-related symptoms, liver function, alpha-fetoprotein, and Child-Pugh score were adjusted for, with no significant differences observed, and subgrouping according to texture analysis proved to be the only factor significantly related to OS. For TTP, subgrouping showed significant differences in the Kaplan-Meier survival curves, but not in the Cox regression. The reason for this observation might be that in the initial screen by Cox regressions, we used survival status (OS) as the event, which probably excluded some parameters related to TTP.

Texture analysis is associated with challenges in image acquisition. In a previous phantom study, texture parameters were demonstrated to be relatively sensitive to tube voltage, but to be independent of the tube current [29]. Additionally, one study showed that hepatic texture features were less sensitive to changes in CT acquisition parameters [30]. Slice thickness is another major determinant of textural parameters [31], with one study revealing that a slice thickness of ≤ 3 mm was optimal for feature grading [32]. Thus, we carefully excluded images outside this criterion in the present study, which might have partly reduced the influence of textural parameter reproducibility in prognostic evaluation.

This retrospective study had some limitations. First, this study included a relatively small sample size. However, in an attempt to control for possible confounding effects, patients with multiple lesions or extrahepatic metastasis were excluded. Second, the retrospective design of this study did not include some potential confounding factors. In particular, the prevalence of comorbidities that might influence liver texture, such as diabetes, alcoholic liver disease, and early cirrhosis was unknown and needs to be assessed in future studies. Further, the possibility of selection bias could not be eliminated. Finally, in this study, all ROIs were manually drawn by the two radiologists rather than by automatic segmentation. However, excellent inter-observer agreement was observed. Future adoption of a more robust algorithm is warranted.

In conclusion, textural variations on baseline CT images might offer more thorough insight for HCC prognosis. Additionally, detailed grouping by wavelet features showed the feasibility of this method in patient stratification. Though further validation is still warranted, texture analysis could potentially be used to inform the LR *vs*. TACE decision-making strategy.

## MATERIALS AND METHODS

### Patients

This study was approved by the Ethics Committee of Guangdong General Hospital. Informed consent was waived due to the retrospective design of the study, and all patient records and information were anonymized and de-identified prior to analysis.

**Figure 4:**
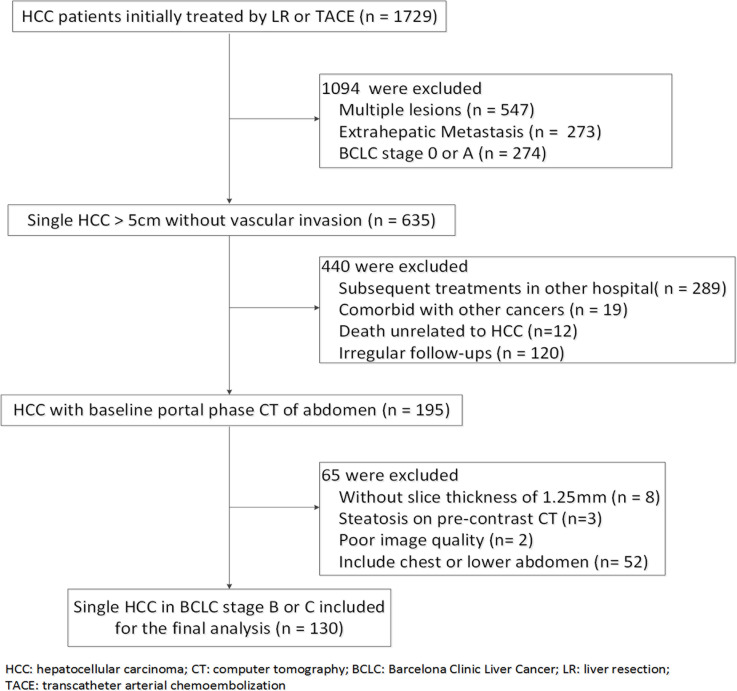
Flowchart for patient inclusion and exclusion

Between September 2009 and December 2014, 130 patients with a single large HCC (> 5cm) initially treated by LR or TACE were enrolled (Figure 4). The time interval between baseline CT and initial treatment was less than 14 days. For BCLC stage C, only patients with branch vascular invasion were included, whereas those with extrahepatic metastasis and main portal vein thrombosis were excluded due to limited efficacy of LR/TACE for these patients. For all enrolled patients (if still alive by the study end date of March 2015), at least a three-year follow-up was required; patients diagnosed after March 2012 without death were excluded from this requirement.

In this study, we employed the BCLC classification instead of TNM, as the BCLC classification is more informative than TNM regarding survival outcomes and therapeutic strategies [2, 3]. In the BCLC classification, disputes exist on the classification of single HCCs > 5 cm without vascular invasion and extrahepatic metastasis as stage A or B [5, 28]. Herein, we used stage AB, as proposed by a previous study [28]. As a result, single HCCs > 5 cm with well-preserved liver function (Child Pugh A-B cirrhosis, and PST < 1) were classified as stage AB (without vascular invasion) and stage C (with vascular invasion). Patients with PST = 1 but without vascular invasion were still classified as stage AB, which was in accordance with two studies [28, 33].

Anatomical or non-anatomical LR was performed with a margin > 10 mm. All TACE procedures in this study were performed by Seldinger's technique, with epirubicin, lobaplatin and lipiodol mixed as the embolic agent.

### Follow-up and endpoint

The follow-up interval was 4-8 weeks, and included routine laboratory tests, chest X-ray and abdominal CT. Additional CT or magnetic resonance imaging was routinely performed if extrahepatic metastasis was suspected.

The primary endpoint was OS, and the secondary endpoint was time to progression (TTP). Disease progression for TACE was defined as an increase of at least 20% in the diameter of a viable target lesion according to the modified Response Evaluation Criteria in Solid Tumors. Disease progression for the LR group was defined as intrahepatic or extrahepatic recurrence.

### CT examination

All baseline images were derived from our picture archiving and communication system. Portal-phase CT of liver was obtained by the same scanner (LightSpeed VCT 64; GE Medical Systems, Waukesha, WI). After administering iopamidol (370 mg of iodine/mL, Iopamiro; Bracco, Milan, Italy), a non-ionic contrast medium, at 1.5 mL/kg (maximum dose, 100 mL) with a double-tube high-pressure syringe at 3.5 mL/s, hepatic imaging acquisition was performed at fixed time points at the portal venous phase at a 70 sec delay. The scan parameters were as follows: 120 kV; automatic mA, 80-500 mA; noise index: 7; pitch/table speed = 0.984/39.37 mm/rot; rotation time, 0.6 s; field of view, 300-450 mm; matrix, 512 mm. A slice thickness of 1.25 mm was routinely reconstructed with soft kernels.

Inspired by one study [34], typical subjective imaging features were analyzed to explore the potential of the conventional image phenotype on HCC prognosis. Six features, including the shape (noninvasive or invasive), capsule (absence, not integral, or integral), corona (negative or positive), mosaic (negative or positive), node-in-node (negative or positive), and enhanced region relative to the entire tumor (< 25%, 25%-50%, 50%-75%, > 75%) were consensus-classified by two radiologists (Figure S3).

### Texture analysis methodology

For each pre-treatment examination, 1.25-mm axial images obtained at the portal venous phase through the largest cross-sectional area of the tumor were selected and transferred to two personal computers for texture analysis. The process of texture analysis comprised three steps: (1) image filtration, (2) wavelet analysis and (3) feature extraction. The first two steps were performed using MATLAB 2014a software (MathWorks Inc., Natick, MA).

(1) Image filtration: Laplacian of Gaussian (LoG) spatial band-pass filters were used to reduce the sensitivity to noise. Filter width and sigma (σ) are the two parameters that characterize LoG filter weighting. Three σ values (0, 1.0, and 1.5) and a single filter-width of σ*5 pixels were used (Table S2). Pixels with attenuation of less than −50 HU were removed. The filtration process produced a series of images displaying textual features at different filters.

(2) Wavelet analysis: The use of the wavelet transform for texture analysis was first proposed by Mallat [35]. This transform provides a robust methodology for texture analysis in different scales. Initially, it decomposes each image and receives its texture by using a series of elemental functions called wavelet and scaling, where “s” governs the scaling and “u” the translation, as follows:

$$\begin{array}{cc} \varphi_{s,u}(x)=\frac{1}{\sqrt{s}}\varphi (\frac{x-u}{s}) & \left(\begin{array}{cc} \text{s}\in R^{+} & u\in R \end{array}\right) \end{array}$$

As a result, the Haar wavelet transform decomposes each original image into nine images with different scales, called trends and fluctuations:

$$\text{Wf}(\text{s},\text{u})=\int_{R}f(x)\frac{1}{\sqrt{s}}\varphi (\frac{x-u}{s})\text{dx}$$

The former are averaged versions of the original image, and the latter contains the high frequencies. Each image is decomposed into 1, 2, or 3 levels and reconstructed in three directions (diagonal, horizontal and vertical).

(3) Texture feature extraction: Two radiologists (Readers 1 and 2, with 5 and 4 years of experience in abdominal CT interpretation, respectively) independently performed textural feature extraction and quantification using ImageJ software (National Institutes of Health, Bethesda, MD). For each reader, a user-defined irregular ROI was drawn manually around the largest cross-sectional tumor outline and copied to the nine derived texture feature maps. Subsequently, the values of the texture features were measured and saved for further analysis.

### Statistical analyses

The Shapiro-Wilk test was applied to assess normality. Differences in patient demographics and characteristics for those undergoing LR or TACE were tested using independent-sample *t*-tests, Mann-Whitney U tests and Chi-square tests. Inter-observer agreement on textual features was evaluated using intraclass correlation coefficients (ICCs) [36].

Patient demographics and subjective imaging features were included for adjustment in the analyses. Univariate Cox regression was used as a preliminary screening of candidate variables. Variables of statistical significance in the univariate analysis (*P* < 0.10) were used as input variables for the subsequent multivariate Cox regression models (Forward: LR method). Textural features at each filter were tested in separate models to assess the independent effects of the CT texture of the primary tumor on OS. Thus, six multivariate models were created (one per group per filter; LR and TACE groups, and three filters). Afterwards, the median values of the independent texture parameters were used to separate patients in the LR and TACE groups for subsequent Kaplan-Meier analysis.

To explore the potential role of texture features in deciding between LR and TACE, patients were first divided into two subgroups according to the identified prognostic markers in the LR and TACE groups. Cox regression for all patients was performed to determine whether subgrouping was an independent factor for OS and TTP. Next, one way-ANOVA or Kruskal-Wallis *H* was used to compare the identified textural parameters among the subgroups. Post hoc multiple comparisons were performed using Bonferroni's correction or Dunnett's T3 test.

The thresholds of the identified factors in Cox regression models were also determined using standard receiver operating characteristic curves. Figure S1 & S2 contain detailed discussions regarding this approach.

All statistical analyses were performed with SPSS 20.0 (IBM SPSS Statistics, Armonk, NY). A two-tailed *P* value of less than 0.05 was considered statistically significant.

## SUPPLEMENTARY MATERIAL FIGURES AND TABLES


