# Supplementary material for: Computed tomography texture analysis to facilitate therapeutic decision making in hepatocellular carcinoma
Source: Oncotarget. 2016 Feb 17;7(11):13248–59. doi: 10.18632/oncotarget.7467 (PMC4914356; doi:10.18632/oncotarget.7467)
Supplement: Supplementary file 1 [file oncotarget-07-13248-s001.pdf]

Computed tomography texture analysis to facilitate therapeutic decision making in hepatocellular carcinoma

Supplementary Material

Table S1. Univariate Analyses of Textural Parameters

**Factors**	**LR**		**TACE**	
	**HR (95% CI)**	***P***	**HR (95% CI)**	***P***
**Filter0**				
**wavelet-1-D**	0.997 (0.956–1.041)	0.905	1.026 (0.950–1.107)	0.519
**wavelet-1-H**	0.993 (0.692–1.029)	0.692	1.028 (0.954–1.107)	0.471
**wavelet-1-V**	0.973 (0.919–1.031)	0.361	1.071 (0.975–1.175)	0.153
**wavelet-2-D**	0.991 (0.920–1.067)	0.807	1.098 (1.000–1.205)	0.049*
**wavelet-2-H**	0.929 (0.845–1.020)	0.123	1.090 (0.995–1.194)	0.065*
**wavelet-2-V**	0.936 (0.861–1.018)	0.124	1.120 (1.012–1.239)	0.029*
**wavelet-3-D**	0.966 (0.875–1.066)	0.488	1.077 (1.000–1.161)	0.052*
**wavelet-3-H**	0.870 (0.750–1.010)	0.067*	1.188 (1.014–1.391)	0.033*
**wavelet-3-V**	0.883 (0.770–1.012)	0.074*	1.188 (1.014–1.391)	0.033*
**Filter1.0**				
**wavelet-1-D**	0.970 (0.826–1.138)	0.707	1.234 (1.019–1.494)	0.032*
**wavelet-1-H**	0.926 (0.807–1.063)	0.277	1.202 (0.966–1.496)	0.099
**wavelet-1-V**	0.929 (0.815–1.059)	0.270	1.242 (1.009–1.529)	0.041*
**wavelet-2-D**	0.984 (0.879–1.102)	0.780	1.166 (1.019–1.335)	0.026*
**wavelet-2-H**	0.836 (0.700–0.998)	0.047*	1.216 (1.005–1.472)	0.044*
**wavelet-2-V**	0.882 (0.766–1.014)	0.079*	1.209 (1.025–1.426)	0.024*

**wavelet-3-D**	0.964 (0.797–1.165)	0.702	1.240 (1.021–1.505)	0.030*
**wavelet-3-H**	0.763 (0.573–1.017)	0.065*	1.431 (1.040–1.970)	0.028*
**wavelet-3-V**	0.720 (0.505–1.026)	0.069*	1.557 (1.039–2.333)	0.032*
**Filter1.5**				
**wavelet-1-D**	0.962 (0.810–1.142)	0.657	1.200 (0.915–1.573)	0.187
**wavelet-1-H**	0.966 (0.831–1.124)	0.656	1.215 (0.907–1.629)	0.192
**wavelet-1-V**	0.904 (0.710–1.151)	0.415	1.452 (0.956–2.206)	0.080*
**wavelet-2-D**	0.905 (0.640–1.281)	0.575	1.663 (1.017–2.721)	0.043*
**wavelet-2-H**	0.750 (0.499–1.128)	0.167	1.632 (0.950–2.802)	0.076*
**wavelet-2-V**	0.626 (0.362–1.081)	0.093*	1.863 (1.013–3.424)	0.045*
**wavelet-3-D**	0.816 (0.353–1.888)	0.635	3.146 (1.102–8.979)	0.032*
**wavelet-3-H**	0.344 (0.101–1.171)	0.088*	4.334 (1.071–17.538)	0.040*
**wavelet-3-V**	0.131 (0.015–1.116)	0.063*	10.440 (1.093–99.685)	0.042*

Note—*factors in the univariate analyses with a *P* <0.10 entered multivariate analyses; LR: liver resection; TACE: transcatheter arterial chemoembolization; HR: hazard ratio.

Table S2. Filter σ Values and the Corresponding Widths of the Filter in Pixels

Sigma(σ)	Filter width(pixels)
o	0
1.0	5
1.5	7.5

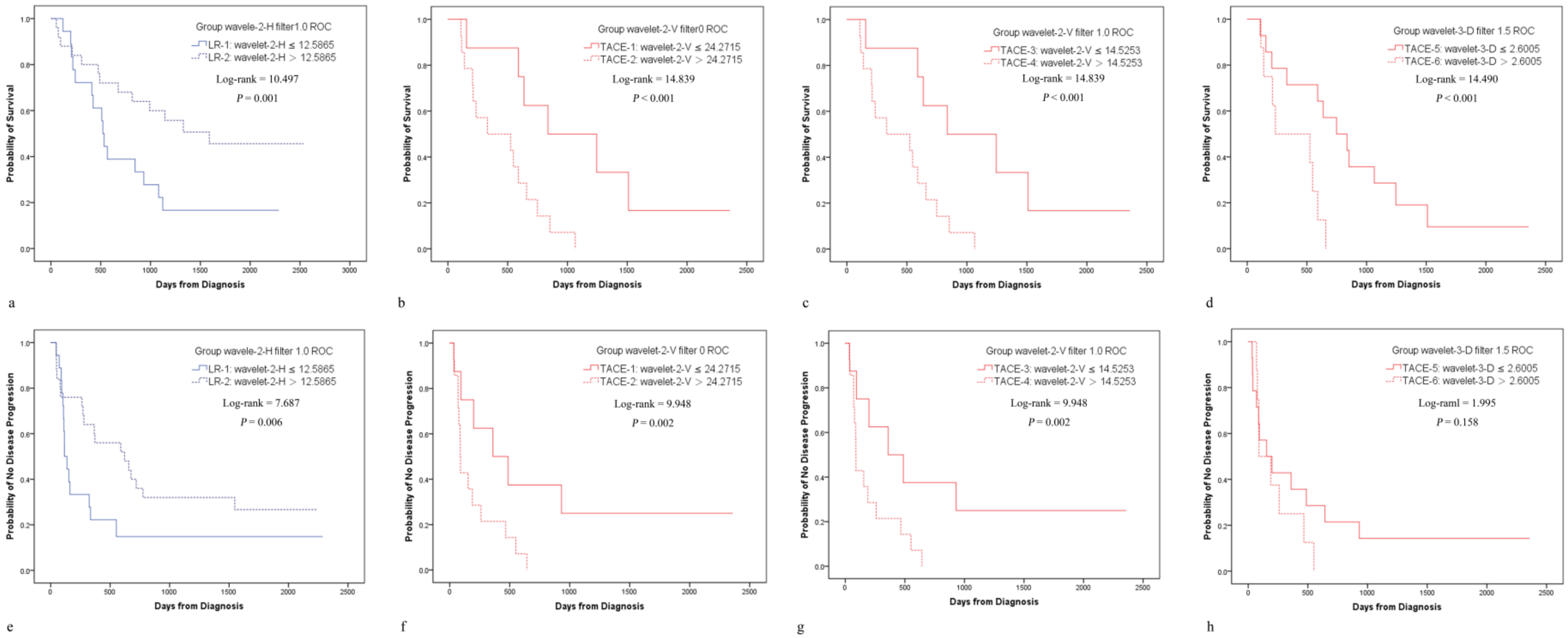

Fig S1: Kaplan-Meier analysis for LR and TACE (separated by the threshold of 3-year survival ROC). In LR, there was a statistical difference in OS (a) and TTP (e), separated by the threshold of wavelet-2-H at filter 1.0. In TACE, there was a statistical difference in OS separated by the threshold of wavelet-2-V (filter 0) (b), wavelet-2-V (filter 1.0) (c) and wavelet-3-D (filter 1.5) (d), respectively. There was a statistical difference

in TTP separated by the threshold of wavelet-2-V (filter 0) (f), wavelet-2-V (filter 1.0) (g), but not wavelet-3-D (filter 1.5) (h). OS= overall survival, TTP= time to progression, LR= liver resection, TACE= transcatheter arterial chemoembolization.

This proved that texture analyses could be used for HCC prognosis, and according to AIC in the main manuscript, wavelet-2-H (filter 1.0) and wavelet-2-V (filter 0) were the best predictors for LR and TACE, respectively.

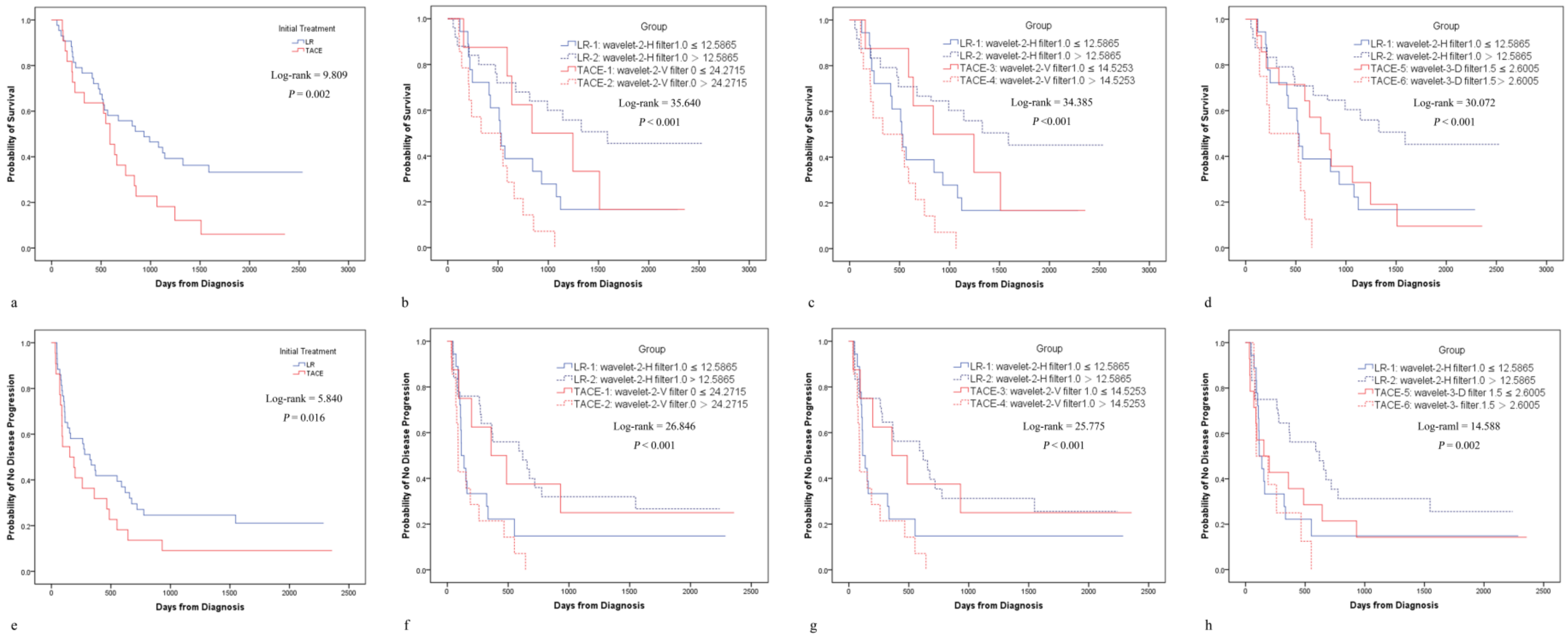

Fig S2. Kaplan-Meier analysis in all the patients (separated by the threshold of 3-year survival ROC). Without subgrouping, OS (a) and TTP (d) had a statistical difference between LR and TACE. LR were subgrouped by the optimal threshold of wavelet-2-H (filter 1.0) (lower: LR-1; higher: LR-2). TACE were sub grouped by the threshold of wavelet-2-V (filter 0) (lower: TACE-1; higher: TACE-2), wavelet-2-V (filter 1.0) (lower: TACE-3; higher: TACE-4) and wavelet-3-D (filter 1.5) (lower: TACE-5; higher: TACE-6).

Among LR-1/2 vs. TACE-1/2 and LR-1/2 vs. TACE-3/4, for OS (b, c), the statistical differences among 4 subgroups were similar to those separated by median; for TTP (f, g), LR - vs. TACE+ had a statistical difference, but LR-1 vs. TACE-2 and LR-1 vs. TACE-4 didn't. The conclusions might be a little different from those in the main text: 1) for TACE-1 or TACE-3, LR might not bring further survival benefit; 2) for TACE-2 or TACE-4, LR might be used instead of TACE to increase survival benefit.

Since separated by wavelet-3-D at filter 1.5, the two TACE groups didn't have statistical difference in both OS and TTP, there was limited meaning to compare the survival of subgroups separated by wavelet-2-H at filter 1.0 (LR -/+) and wavelet-3-D at filter 1.5 (TACE). But among LR-1/2 and TACE-5/6, OS (d) had a statistical difference between some pairwise comparisons: LR-1 vs. TACE-6, LR-2 vs. TACE-5, LR-2 vs. TACE-6; but not between LR-1 vs.TACE-5.TTP (h) had a statistical difference between some pairwise comparisons: LR-2 vs. TACE-5, LR-2 vs. TACE-6, but not between others: LR-1 vs.TACE-5 and LR-1 vs. TACE-6. This could lead to similar conclusions in the main text: 1) for LR-2， LR was the best choice; 2) for LR-1, TACE might be used instead in consideration of LR-related risks and complications; 3) for TACE-6, LR might be used instead of TACE to increase survival benefit.

By comparing the results in Fig 1 vs. Fig S1, Fig 2 vs. Fig S2, we concluded that when separated by the threshold of ROC (appendix), the

survival curves tended to have more significant statistical difference. But the cost was the possibility of unbalance among subgroups, which could make the conclusion only suitable for the minority. When separated by median value (main document), the survival curve tended to have less significant statistical difference (see the results of wavelet-3-D at filter 1.5 for TACE). But the advantage was that patients would be equally distributed, which ensured the conclusion suitable for halves. So when sensitivity (discrimination) was the primary consideration, threshold was preferable, whereas when universality was more important, median was preferable.

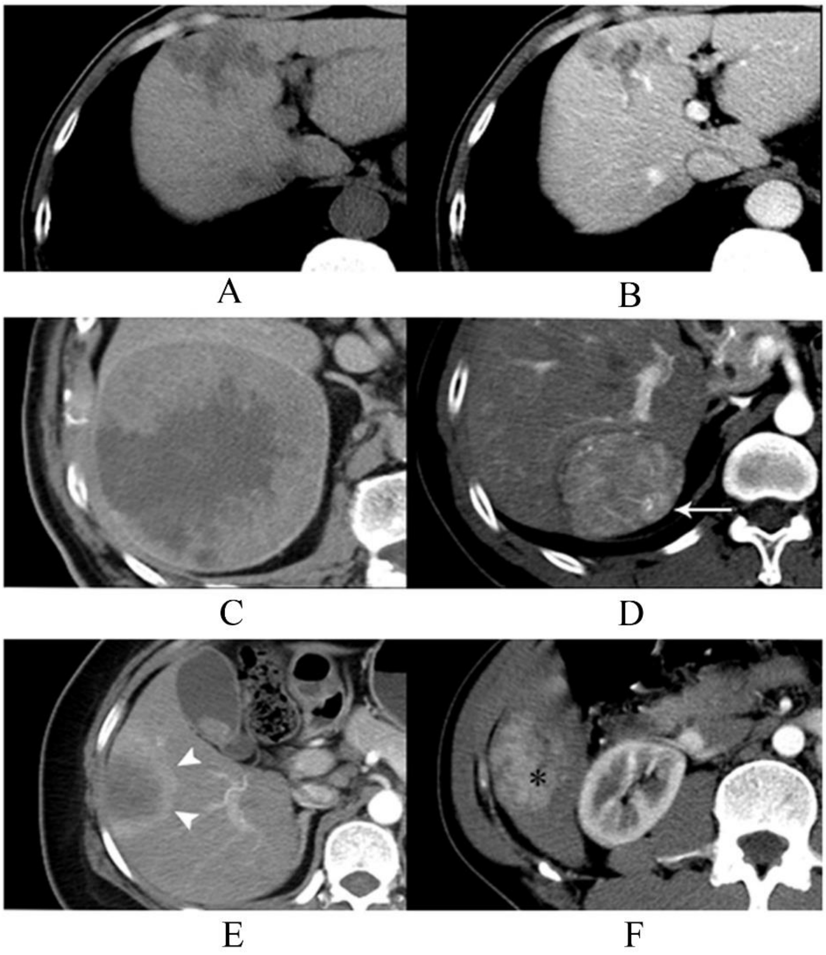

Fig S3. Illustrations for shape, capsule, nodule-in-nodule, corona and mosaic architecture. HCC was identified as invasive at plain scan (A) and portal venous phase (B), which was in agreement with the pathologic concept of “exogenous growth”. (C) CT image at delayed phase showed capsule appearance. As opposed to corona enhancement, which fades in the venous phases (not shown), capsule appearance manifests as progressively enhancing rim. (D) CT image at hepatic arterial phase with HCC show nodule-in-nodule architecture (arrow). Arterial phase hyperenhancement and venous phase washout appearance of inner nodule suggest progressed HCC arising within more well-differentiated parent nodule. (E) Note circumferential enhancement (arrowheads) of variable thickness in the liver parenchyma around the

mass in the hepatic arterial phase. Corona enhancement is thought to represent the area of liver parenchyma receiving venous drainage from progressed HCC and to be a frequent site of satellite metastases. (F) CT image acquired in hepatic arterial phase showed a heterogeneous mass with mosaic architecture (*).Mosaic architecture refers to the presence within a mass of randomly distributed internal nodules or compartments differing in enhancement attenuation, intensity, shape, size and often separated by fibrous separations. [1]

1. Choi JY, Lee JM and Sirlin CB. CT and MR imaging diagnosis and staging of hepatocellular carcinoma: part II. Extracellular agents, hepatobiliary agents, and ancillary imaging features. Radiology. 2014; 273(1):30-50.
